# Feasibility and Safety of Intraoperative Abdominal Warming During Spinal Surgery in the Prone Position: A Pilot Study

**DOI:** 10.7759/cureus.116393

**Published:** 2026-09-16

**Authors:** Masato Ishii, Beniko Itokawa

**Affiliations:** 1 Department of Emergency Medicine, Akita Red Cross Hospital, Akita, JPN; 2 Department of Nursing, Faculty of Nursing, The Japanese Red Cross Tohoku College of Nursing, Akita, JPN

**Keywords:** abdominal warming, core body temperature, forced-air warming, inadvertent perioperative hypothermia, patient safety, pilot study, prone position surgery, spine surgery, underbody blanket

## Abstract

Background: Inadvertent perioperative hypothermia caused by general anesthesia leads to several complications, including shivering, coagulopathy, surgical site infections, and delayed wound healing. Prone positioning for spine surgery severely restricts the available body surface area for warming, complicating intraoperative temperature management. This study investigated a novel approach utilizing the anatomical space beneath the chest and abdomen created by a standard four-point bolster support to maintain intraoperative core body temperature using standard pediatric underbody forced-air warming blankets.

Materials and methods: This prospective, single-arm pilot study enrolled six adult patients undergoing elective spinal surgery in the prone position under general anesthesia at Akita Red Cross Hospital, Akita, Japan. No active preoperative warming was performed. A pediatric underbody forced-air warming blanket (SWU-2009; ICU Medical Inc., San Clemente, CA, USA) was pre-positioned in the suspended thoracoabdominal space of the four-point support and operated at 40 °C. Core body temperature (monitored via a temperature-sensing urinary catheter) and skin condition were recorded from induction to the end of surgery.

Results: Six patients (mean age: 66.8 ± 16.7 years, mean surgical duration: 230.2 ± 55.5 minutes) were analyzed. The median core temperature was 36.5 °C (interquartile range (IQR): 36.4 °C-36.5 °C) at induction, 36.6 °C (IQR: 36.4 °C-36.9 °C) at incision, and 37.0 °C (IQR: 36.7 °C-37.1 °C) at the end of surgery. Although transient mild hypothermia (< 36.0 °C) occurred in two patients, both recovered to normothermia within 90 minutes. No patient developed redness, blisters, or thermal burns.

Conclusions: Abdominal warming utilizing the suspended space of a four-point bolster support was feasible to implement without occurrences of skin-related complications, demonstrating potential to contribute to intraoperative temperature stabilization. While adequately powered comparative trials are required to definitively evaluate its clinical efficacy, this approach provides preliminary insights into practical thermal management in standard operating rooms.

## Introduction

Redistribution hypothermia associated with general anesthesia induces various intraoperative and postoperative complications, including shivering, delayed emergence, coagulopathy, infection due to impaired immune function, cardiovascular events, and delayed wound healing, all of which significantly affect patient recovery and postoperative management [1, 2]. Notably, even mild hypothermia of less than 1 °C has been reported to inhibit platelet function and coagulation factor activity, thereby increasing intraoperative blood loss by approximately 16% and raising the relative risk of requiring blood transfusions by approximately 22% [3].

Temperature management is particularly challenging during spinal surgery performed in the prone position because the anatomical areas available for active warming are severely restricted. Currently, the standard practice for maintaining the prone position in spinal surgery involves a combination of a conventional operating table and a four-point bolster support system. While forced-air warming devices are widely used for intraoperative thermoregulation, active warming options in the prone position remain limited. To date, no previous studies have investigated selective cutaneous warming of the anatomically suspended abdominal space created by a four-point bolster support during prone spinal surgery.

In Japan, previous studies have investigated warming methods such as forced-air warming devices and upper/lower extremity coverage [4,5,6]. There is also an increasing number of reports indicating that preoperative and surface warming are effective in preventing redistribution hypothermia [7, 8], findings that underscore the importance of nursing interventions from the preoperative preparation stage. However, investigations into the effects of abdominal warming have been limited to studies utilizing gas warming and humidifying devices in laparoscopic surgery [9], with virtually no research focusing on the direct effects of cutaneous warming or its feasibility in clinical practice. Furthermore, intraoperative hypothermia progresses due to a complex interplay of initial redistribution hypothermia, radiation, convection, and evaporation. Spinal surgery presents a unique challenge in that extensive skin exposure is unavoidable until prone positioning is complete, making patients highly susceptible to heat loss.

Given this background, the present approach focused on the anatomical space created beneath the chest and abdomen by the use of a four-point bolster support. While this space was conventionally considered a factor promoting heat loss [4], applying active forced-air warming directly into this suspended, non-weight-bearing area was hypothesized to offer a practical method that eliminates procedural complexities, such as repositioning and smoothing blankets under pressure points, inherent in conventional prone warming techniques. Theoretically, simplifying this positioning and warming workflow could reduce the duration of skin exposure during positioning, thereby mitigating heat loss. However, before considering the broader clinical implementation of this approach, it is imperative to first evaluate whether it is feasible to execute and capable of contributing to core temperature stabilization without compromising skin integrity.

Because this intervention represents a novel approach that has not been investigated in prior literature, the primary aim of this pilot study was to evaluate the feasibility and safety (specifically the prevention of thermal and pressure skin injuries) of selective abdominal warming in the prone position. The secondary aim was to observe preliminary intraoperative core temperature trajectories to provide baseline observations for future comparative investigations.

## Materials and methods

Study design and ethical considerations

This study was a prospective, single-arm pilot trial designed to evaluate the safety and feasibility of a novel abdominal warming strategy during prone spinal surgery. The study protocol was approved by the Nursing Research Ethics Committee of Akita Red Cross Hospital, Akita, Japan (approval number: 5-B-1). Prior to enrollment, the purpose, methods, and clinical significance of the study, as well as the risks and benefits, were explained both verbally and in writing to all eligible patients during preoperative visits by operating room nurses. Written informed consent was obtained from all participants. Patient privacy was strictly protected, and participants were assured of their right to withdraw from the study at any time without any clinical penalty. Codes (Patients A-F) were assigned to all patients to ensure confidentiality and anonymity of the patients.

Participants

Adult patients scheduled to undergo elective spine surgery in the prone position under general anesthesia at Akita Red Cross Hospital between January and June 2023 were enrolled. The inclusion criteria were patients classified as American Society of Anesthesiologists physical status (ASA-PS) I or II who provided written informed consent.

To eliminate confounding factors that could independently affect thermoregulation and temperature trajectories, the exclusion criteria were established as follows: (1) patients with pre-existing endocrine or metabolic disorders (e.g., uncontrolled diabetes, thyroid disorders); and (2) patients who received active preoperative warming (prewarming) in the preoperative holding area.

Sample size

As this was an initial pilot study focusing on the feasibility and safety of a novel warming intervention, a formal sample size calculation based on statistical hypothesis testing was not performed. A recruitment window from January to June 2023 was established with a prospective target of up to 10 patients to observe preliminary physiological trends and identify potential procedural risks. The study protocol dictated that patient recruitment would conclude strictly upon the expiration of this six-month period, regardless of whether the maximum target of 10 patients had been reached. While this limited sample size restricts the generalizability of the findings, it provides preliminary baseline data that may inform the design of future comparative trials.

Intervention protocol

Prior to the patient's arrival in the operating suite, the forced-air warming unit (LEVEL1 EQ-500; ICU Medical Inc., San Clemente, CA, USA) and the pediatric underbody warming blanket (SWU-2009, 65 × 115 cm; ICU Medical Inc., San Clemente, CA, USA) were inspected for safety. The operating room ambient temperature was maintained at a constant 24 °C-26 °C before anesthesia induction. The pediatric warming blanket was pre-positioned in the suspended anatomical space between the bolsters of the four-point support system (Figure 1).

**Figure 1:**
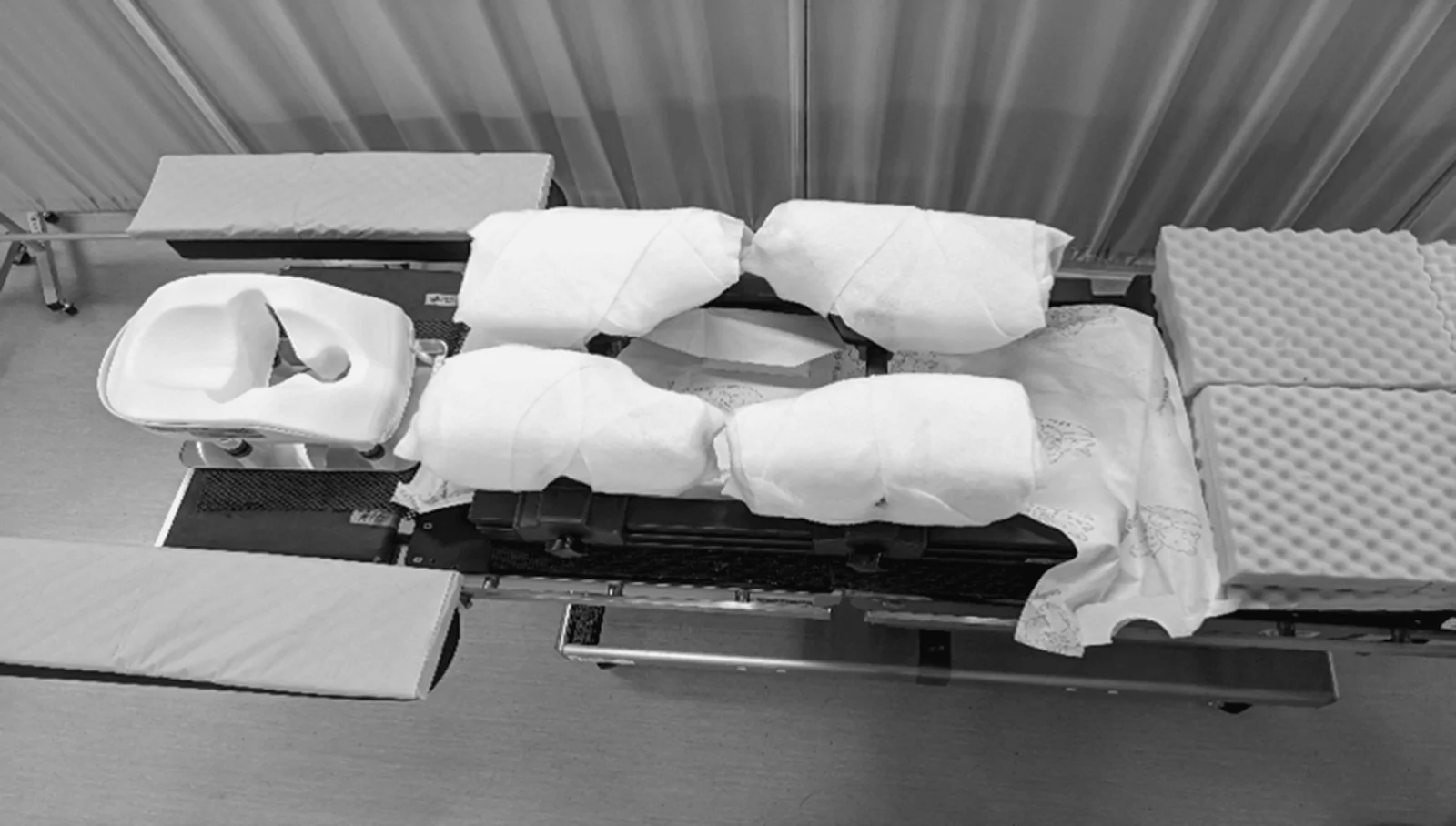
Preoperative setup of the warming blanket beneath the four-point bolster system. A standard pediatric underbody forced-air warming blanket is pre-positioned in the suspended anatomical space created by the four-point bolster supports on a conventional operating table before patient arrival. Image credits: This image has been photographed by the authors.

After placement, the device was briefly activated to confirm proper airflow and warming without abnormalities. To facilitate safe patient transfer and position conversion, the blanket and hose were then temporarily disconnected. The patients were covered only with standard cotton blankets in the preoperative area. Active forced-air warming was not initiated preoperatively; patients who requested prewarming were excluded from the study.

Following standard anesthesia induction and endotracheal intubation, a temperature-sensing urinary catheter (Lubri-Sil I.C. Complete Care Temperature-Sensing Foley Catheter Tray; Bard Medical, Covington, GA, USA) was inserted to monitor bladder temperature, which served as the core body temperature. The patient was then carefully turned into the prone position and secured on the standard operating table using the four-point bolster support. Once positioning was finalized, the pre-positioned SWU-2009 pediatric warming blanket was connected to the LEVEL1 EQ-500 forced-air warming hose (Figure 2).

**Figure 2:**
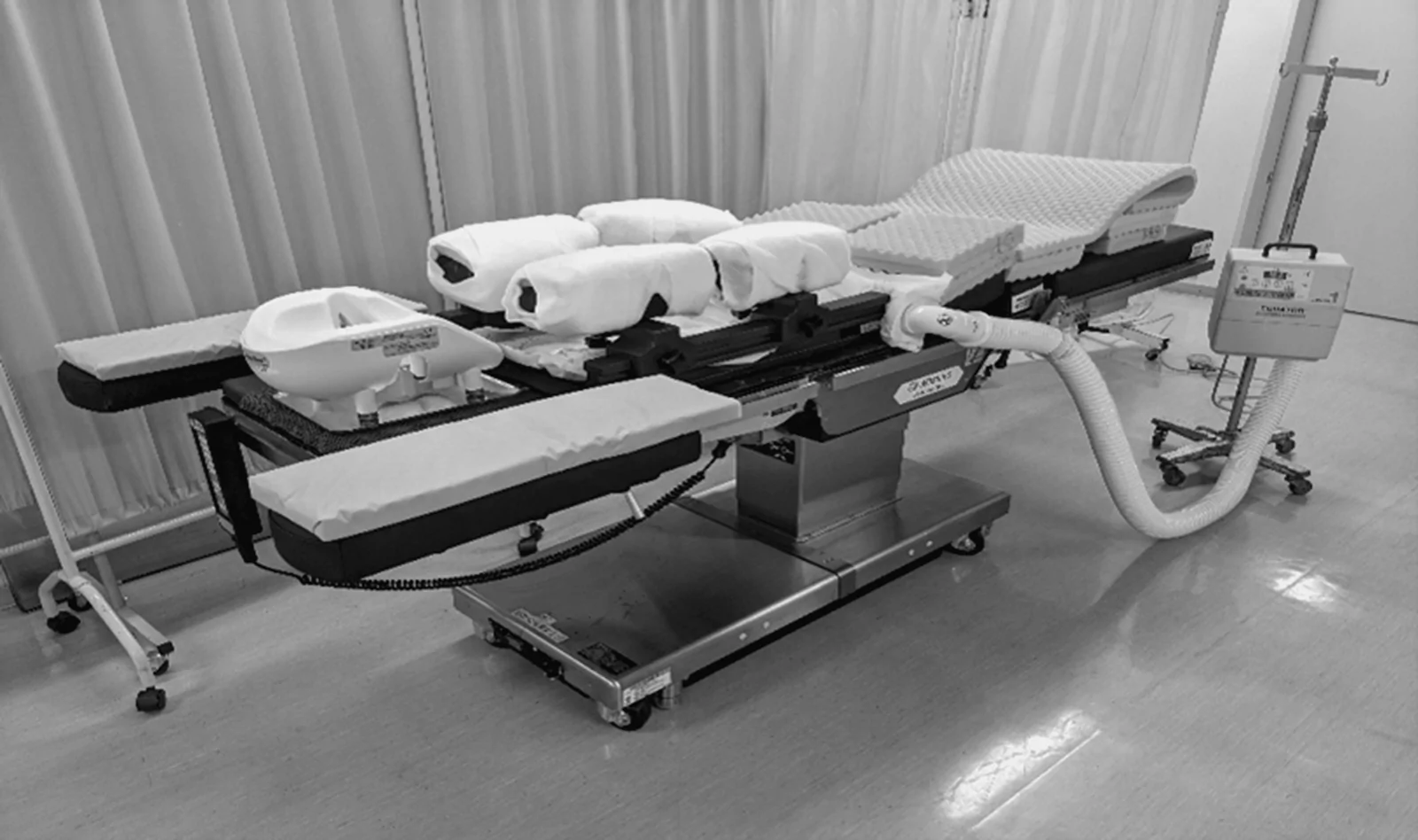
Overall view of the connected abdominal warming system for prone spinal surgery. The fully assembled system, showing the forced-air warming unit connected to the pre-positioned underbody blanket. This setup is designed to deliver heat directly to the patient's suspended abdominal region. Image credits: This image has been photographed by the authors.

The forced-air warming device was turned on immediately after the surgical incision was made, and the temperature setting was maintained at a constant 40 °C throughout the procedure. The forced-air warming device utilized in this study allowed only temperature adjustment, with no variable airflow settings available. After activation, although there were areas of partial contact between the inflated blanket and the patient's abdominal skin, it was strictly ensured that the hose did not come into direct contact with the skin. At the end of the surgery, the warming device was turned off and removed. The final temperature measurement was recorded immediately before the patient was turned back to the supine position.

Measurements and outcomes

Patient demographics, including age, body mass index (BMI), and body surface area (BSA, calculated using the Du Bois formula), were extracted from the electronic medical records. Intraoperative variables, including surgical duration, blood loss, and total intravenous fluid volume, were obtained from the surgical and anesthetic records. Core body temperature (bladder temperature) was measured using temperature-sensing urinary catheters that were within their manufacturer-guaranteed expiration dates, and the monitors used to display the temperatures were regularly inspected and calibrated by clinical engineers. The temperatures were recorded every 30 minutes from the beginning of anesthesia to the end of surgery.

To evaluate safety, skin condition at the abdominal warming site was monitored. Operating room nurses visually inspected the patient's thighs and abdomen at one-hour intervals to check for any signs of thermal injury or localized pressure, including redness, swelling, blisters, or burns. A strict safety protocol was established. If any abnormal skin changes were observed, the forced-air warming device was to be shut down immediately, and the anesthesiologist and primary surgeon were to be notified.

Statistical analysis

Due to the small sample size and potential non-normal distribution of the data, core body temperature trajectories are described as median and interquartile range (IQR). For exploratory analysis, core body temperature at different intraoperative time points was compared using the Friedman test, and post-hoc comparisons were performed using the Wilcoxon signed-rank test with Bonferroni correction. A p-value of less than 0.05 was considered statistically significant. However, given that this pilot study was conducted on a small sample (N = 6), the statistical power is low, increasing the risk of Type II errors. Therefore, these statistical tests were interpreted strictly as descriptive auxiliary markers. All statistical analyses were performed using R version 4.6.0 (R Foundation for Statistical Computing, Vienna, Austria).

## Results

Patient demographics and surgical variables

During the predetermined six-month study window (January to June 2023), six patients met the eligibility criteria and consented to participate. All six patients provided written informed consent and were successfully enrolled. None of the six patients declined participation, and no patients were excluded for meeting exclusion criteria (such as pre-existing endocrine disorders or requesting active preoperative warming). Furthermore, there were zero post-enrollment dropouts or exclusions. Because patient recruitment ceased strictly upon the conclusion of the six-month study period, the final analyzed cohort consisted of these six patients. The demographic and clinical characteristics of the study population are summarized in Table 1.

**Table 1:** Patient demographics and surgical variables

Parameter	Mean±SD
Age (years)	66.8±16.7
Body surface area (m^2^)	1.75±0.32
Body mass index (kg/m^2^)	26.8±4.4
Surgical duration (min)	230.2±55.5
Estimated blood loss (mL)	172.5±130.2
Intravenous fluid volume (mL)	1425.0±302.1

Intraoperative core temperature trajectories

The chronological changes in patients' core body temperature (bladder temperature) are presented in Table 2. The median temperature was 36.5 °C (IQR: 36.4 °C-36.5 °C) at anesthesia induction and 36.6 °C (IQR: 36.4 °C-36.9 °C) at the beginning of surgery. At 30, 60, and 90 minutes after surgical incision, the median temperatures were 36.4 °C (IQR: 36.0 °C-36.9 °C), 36.3 °C (IQR: 36.2 °C-36.9 °C), and 36.3 °C (IQR: 36.1 °C-36.9 °C), respectively. The median temperature stabilized and rose to 36.4 °C (IQR: 36.2 °C-36.9 °C) at 120 minutes and reached 37.0 °C (IQR: 36.7 °C-37.1 °C) at the end of surgery (Figure 3).

**Table 2:** Chronological changes in intraoperative core body temperature All core body temperatures were measured via a temperature-sensing urinary bladder catheter and are expressed in degrees Celsius (°C). N = 6. Codes (Patients A–F) were assigned to all patients to ensure confidentiality and anonymity of the patients. IQR: interquartile range; min: minutes

Patient	Anesthesia Induction	Surgical Incision	30 Min	60 Min	90 Min	120 Min	End of Surgery
A	36.4	36.6	36.4	36.3	36.4	36.4	36.6
B	36.4	36.5	36.3	36.2	36.2	36.2	36.8
C	36.5	36.3	35.6	36.2	36.0	36.3	36.6
D	36.5	36.3	35.9	35.9	36.0	36.1	37.1
E	36.2	37.0	37.0	37.1	37.1	37.1	37.1
F	37.2	37.5	37.4	37.1	37.0	37.0	37.2
Median	36.5	36.6	36.4	36.3	36.3	36.4	37.0
IQR	36.4–36.5	36.4–36.9	36.0–36.9	36.2–36.9	36.1–36.9	36.2–36.9	36.7–37.1

**Figure 3:**
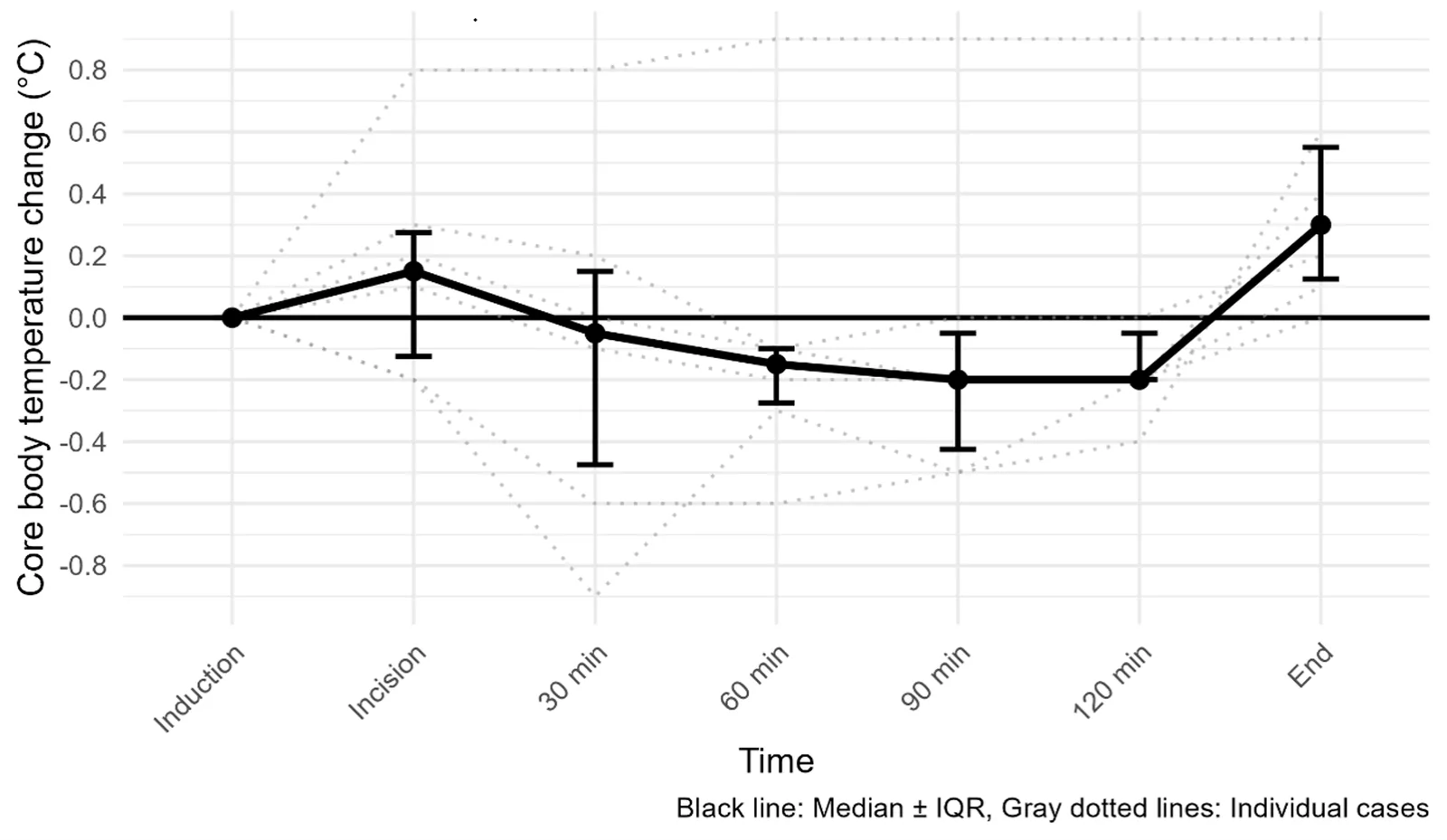
Chronological changes in perioperative core body temperature. The solid black line represents the median core body temperature with error bars indicating the interquartile range (IQR). The gray dotted lines represent the temperature trajectories of individual patients (N = 6). Core body temperature was measured via a temperature-sensing urinary bladder catheter. min: minutes

Continuous urinary drainage was strictly monitored throughout the procedure. All six patients maintained continuous urine output (> 0.5 mL/kg/h), with no episodes of oliguria or catheter obstruction.

Perioperative hypothermia (defined as core body temperature < 36.0 °C) [10, 11] was observed in two of the six patients (Patients C and D) during the early phase of surgery. For Patient C, the core temperature declined to 35.6 °C at 30 minutes after surgical incision and recovered to 36.2 °C at 60 minutes. For Patient D, the core temperature decreased to 35.9 °C at 30 and 60 minutes after incision and recovered to 36.0 °C at 90 minutes. Both patients remained normothermic (≥ 36.0 °C) for the remainder of the procedure. At the end of the surgery, no patient had a core body temperature below their baseline level recorded at anesthesia induction.

Exploratory statistical analysis confirmed these clinical observations and revealed a statistically significant change in core body temperature over the surgical time course (Friedman test, p = 0.018). However, post-hoc pairwise comparisons using the Wilcoxon signed-rank test with Bonferroni correction revealed no statistically significant differences between the baseline (anesthesia induction) and any subsequent intraoperative time points up to 120 minutes (all adjusted p-values = 1.00). This indicates that while physiological temperature shifts occurred, the core temperature did not significantly deviate or dangerously decline from the baseline at any specific phase, demonstrating stable thermoregulation throughout the procedure (Table 3).

**Table 3:** Statistical comparison of core body temperature against the baseline (anesthesia induction) *Adjusted p-values for pairwise comparisons against baseline were calculated using the Wilcoxon signed-rank test with Bonferroni correction. The overall Friedman test indicated a significant chronological change (p = 0.018). ns: not significant; min: minutes

Time Point	Unadjusted p-value	Adjusted p-value*	Significance
Surgical incision	0.32	1	ns
30 min	0.7	1	ns
60 min	0.76	1	ns
90 min	0.71	1	ns
120 min	0.93	1	ns

Safety and skin assessment

Visual inspections of the abdomen and thighs conducted at one-hour intervals revealed no skin abnormalities in any patient. There were no occurrences of redness, swelling, blistering, or thermal burns at the abdominal warming site.

## Discussion

This prospective pilot study investigated the feasibility and preliminary thermal trends of a novel abdominal warming strategy during elective spine surgery in the prone position. By placing a standard pediatric underbody forced-air warming blanket in the suspended, non-weight-bearing space created by a four-point bolster support, intraoperative thermal trajectories were evaluated in six patients. Normothermia was preserved in four patients, while two experienced transient, mild hypothermia during the initial surgical phase. Furthermore, no thermal or pressure-related skin injuries were observed. These preliminary findings indicate that this positioning-based approach was feasible to implement, although its comparative clinical effectiveness and utility warrant further validation in larger trials.

The clinical significance of maintaining normothermia utilizing this approach must be interpreted within the broader context of perioperative quality improvement. Recently, the Enhanced Recovery After Surgery (ERAS) Society strongly recommended active warming in their consensus guidelines for lumbar spinal fusion to minimize surgical complications [12]. In extensive spinal surgeries, surgical site infections (SSIs) can be catastrophic. A landmark randomized trial established that perioperative normothermia significantly reduces the incidence of SSIs and shortens hospitalization [13], while recent systematic evidence continues to highlight the critical role of temperature management in mitigating infection risks, particularly in high-risk cohorts and specific surgical populations [14]. By introducing a practical and feasible method to achieve this standard of care, the presented abdominal warming approach aligns seamlessly with global ERAS protocols and the overarching goal of preventing severe postoperative complications.

An important physiological and safety advantage of this technique is that the abdomen is completely suspended and does not bear patient weight when positioned on standard four-point bolsters. External pressure applied to the skin during active warming can impair localized microvascular blood flow, posing a risk of thermal accumulation and localized burn injuries. Warming a suspended, non-weight-bearing region like the abdomen preserves microvascular perfusion, thereby minimizing the risk of localized thermal injuries. This theoretical advantage is supported by the safety results, as no patient developed redness, swelling, or burns.

Despite the continuous active warming, two patients (Patients C and D) experienced transient, mild hypothermia (< 36.0 °C) during the first 60 minutes after incision. This early core temperature decline is primarily caused by core-to-peripheral heat redistribution, a physiological consequence of anesthetic-induced vasodilation that underscores the importance of intraoperative warming [15]. However, both patients recovered to normothermia (≥ 36.0 °C) within 90 minutes. This trajectory suggests that this abdominal warming approach may have contributed to mitigating the initial redistribution-induced temperature drop and stabilizing the core temperature in the later phases of surgery.

In addition to the observed thermal stability, this warming strategy offers practical advantages for perioperative workflow. Traditional prone underbody blankets must be placed and meticulously smoothed over the supports after patient positioning. This process is often technically difficult, extends the time patients are exposed to cold ambient temperatures, and increases the risk of wrinkle-induced medical device-related pressure injuries. In contrast, the present technique pre-positions a standard pediatric blanket in the suspended space of the four-point support before patient arrival. This eliminates the need for adjustments after positioning, which may reduce patient exposure to cold ambient temperatures and streamline positioning procedures.

This pilot study has several limitations that warrant acknowledgment. First, the sample size was small (N = 6), which limits the statistical power of the exploratory findings and increases the risk of Type II errors. Second, this study lacked a control group (e.g., non-warmed patients or patients warmed using traditional methods), which prevents direct comparative efficacy analysis. Third, other perioperative variables that could influence thermoregulation, such as continuous operating room temperature logs, the use of warmed intravenous or irrigation fluids, or individual patient metabolic rates, were not controlled for or documented. Finally, regarding temperature monitoring, the anatomical proximity between the active warming site and the temperature probe must be carefully considered. For instance, Yoo et al. utilized nasopharyngeal temperature probes, which are anatomically close to their upper-body warming site, potentially leading to an overestimation of core temperatures due to localized heat conduction [16]. Similarly, in the present study, temperature-sensing urinary catheters were utilized; however, because the abdominal surface directly below the urinary bladder was actively warmed in the prone position, the measurements may also be susceptible to localized conductive heat transfer. Furthermore, while maintaining adequate urine flow is established to significantly enhance the accuracy and precision of bladder thermometry in reflecting central core temperature [17], bladder temperature is, nonetheless, classified as a near-core monitoring site that remains susceptible to artifacts from surrounding tissue temperatures [18]. In the prone position, because the anterior abdominal wall directly adjacent to the urinary bladder was actively heated, localized conductive heat transfer to the catheter thermistor cannot be entirely ruled out, even in the presence of continuous urine drainage.

Therefore, as the present findings offer preliminary insights into the potential of this warming method, future studies utilizing concurrent core temperature measurements at other anatomical sites are warranted to validate its true clinical efficacy.

## Conclusions

In this pilot study, abdominal warming was feasible to implement without occurrences of skin-related complications, demonstrating potential to contribute to intraoperative temperature stabilization. However, given the limited sample size and lack of a control group, these preliminary findings warrant cautious interpretation. Future randomized controlled trials comparing this approach with standard warming modalities are required to definitively establish its comparative clinical efficacy and safety.
